# CeO_2_-Blended Cellulose Triacetate Mixed-Matrix Membranes for Selective CO_2_ Separation

**DOI:** 10.3390/membranes11080632

**Published:** 2021-08-17

**Authors:** Chhabilal Regmi, Saeed Ashtiani, Zdeněk Sofer, Zdeněk Hrdlička, Filip Průša, Ondřej Vopička, Karel Friess

**Affiliations:** 1 Department of Physical Chemistry, University of Chemistry and Technology, Technická 5, 16628 Prague, Czech Republic; jamalias@vscht.cz (S.A.); vopickao@vscht.cz (O.V.); 2 Department of Inorganic Chemistry, University of Chemistry and Technology, Technická 5, 16628 Prague, Czech Republic; zdenek.sofer@vscht.cz; 3 Department of Polymers, University of Chemistry and Technology, Technická 5, 16628 Prague, Czech Republic; hrdlickz@vscht.cz; 4 Department of Metals and Corrosion Engineering, University of Chemistry and Technology, Technická 5, 16628 Prague, Czech Republic; prusaf@vscht.cz

**Keywords:** greenhouse gas, composite membrane, inorganic fillers, gas separation

## Abstract

Due to the high affinity of ceria (CeO_2_) towards carbon dioxide (CO_2_) and the high thermal and mechanical properties of cellulose triacetate (CTA) polymer, mixed-matrix CTA-CeO_2_ membranes were fabricated. A facile solution-casting method was used for the fabrication process. CeO_2_ nanoparticles at concentrations of 0.32, 0.64 and 0.9 wt.% were incorporated into the CTA matrix. The physico-chemical properties of the membranes were evaluated by SEM-EDS, XRD, FTIR, TGA, DSC and strain-stress analysis. Gas sorption and permeation affinity were evaluated using different single gases. The CTA-CeO_2_ (0.64) membrane matrix showed a high affinity towards CO_2_ sorption. Almost complete saturation of CeO_2_ nanoparticles with CO_2_ was observed, even at low pressure. Embedding CeO_2_ nanoparticles led to increased gas permeability compared to pristine CTA. The highest gas permeabilities were achieved with 0.64 wt.%, with a threefold increase in CO_2_ permeability as compared to pristine CTA membranes. Unwanted aggregation of the filler nanoparticles was observed at a 0.9 wt.% concentration of CeO_2_ and was reflected in decreased gas permeability compared to lower filler loadings with homogenous filler distributions. The determined gas selectivity was in the order CO_2_/CH_4_ > CO_2_/N_2_ > O_2_/N_2_ > H_2_/CO_2_ and suggests the potential of CTA-CeO_2_ membranes for CO_2_ separation in flue/biogas applications.

## 1. Introduction

The separation of CO_2_ from other light gases has been practiced for decades. The significance of this process is directly linked to the importance of CO_2_ as a major anthropogenic greenhouse gas responsible for climate change [1]. Polymer membrane-based gas separation has been considered a promising technology due to advantages such as low energy consumption, operational simplicity, environmental friendliness and high efficiency compared to other gas separation techniques [2]. Despite advancements in the design of novel membrane materials with improved gas separation performance, polymers still play a crucial role in the current gas separation field due to their easy processability and solid mechanical strength, which lay the foundation for their industrial deployment [3]. However, due to the trade-off between permeability and selectivity, it is impracticable to prepare a polymer membrane with simultaneously elevated permeability and selectivity [4]. Permeability can be improved by increasing the polymer’s free volume or using structural features to alter the interchain polymer spacing. However, this approach is unable to improve selectivity [5]. Improving both permeability and selectivity above the Robeson upper bound is desirable but challenging to achieve using a neat polymeric membrane.

Several approaches, including the development of inorganic, isoporous, mixed-matrix and aquaporin membranes for either surface modification or transport facilitation, have been proposed to exceed the upper-bound performance of membranes [6]. Among them, mixed-matrix membranes (MMMs), consisting mainly of polymer and inorganic materials, have been recognized as an essential alternative to overcome the trade-off between permeability and selectivity and bridge the gap with inorganic membranes [7]. Emerging hybrid MMMs, possessing a plethora of unique properties, are synthesized by inserting different inorganic fillers into the polymer matrix [8]. MMMs combine the advantages of both inorganic particles and polymer membranes, integrating the improved separation capabilities of filler particles with the polymer’s good processability and mechanical properties [9]. Moreover, the bottom line for the gas separation performance of MMMs is determined by the nature of the polymer phase used. The ultimate performance enhancement of MMMs is thus determined by the characteristics of the inorganic fillers used (chemical structure, surface chemistry, particle size distribution and aspect ratio) [10]. Inorganic fillers offer efficient ways to tailor pore sizes and shapes and achieve a narrow pore size distribution, potentially leading to high permeability and/or high selectivity [11]. Furthermore, other advantages of inorganic fillers include the enhancement of permeability/selectivity due to the intrinsic molecular transport channels provided by fillers to the bulk polymer phase [7,12], systematic manipulation of the molecular packing of polymer chains and the creation of more free volume for molecular diffusion [13,14]. Functional groups decorated on the filler surface or within filler pores can improve interfacial contact or provide new functions to membranes [15]. Numerous novel inorganic materials with different effects on the gas transport properties of polymeric membranes have been identified. Substantial improvements in gas separation performance have been observed with inorganic fillers, such as carbon and carbon-based oxides [16] and zeolites [17]. Nonporous fillers such as silica [18], clay particles [19] and metal oxides [9,20] have also had beneficial effects on the gas separation performance. The identification of new inorganic fillers is therefore crucial for the continued development of MMMs.

CeO_2_, a rare-earth-metal oxide, was explored as a potential filler material for Nafion in the composition of MMMs, especially for fuel cell applications [21,22]. Similarly, CeO_2_ nanoparticles embedded in a thin-film nanocomposite membrane with a polyethersulfone porous support were used for water treatment [23]. Furthermore, the photocatalytic activity of cellulose acetate nanoceria/Pt hybrid mats was studied for efficient wastewater treatment applications [24]. Moreover, CeO_2_ nanoparticles have great potential as nanofillers due to their low production costs, high surface area, unique acid–base and redox properties and ability to rapidly transform between +3 and +4 oxidation states of Ce [25]. However, to date, CeO_2_ nanoparticles have not been reported for the development of MMMs for gas separation applications.

**Figure 1:**
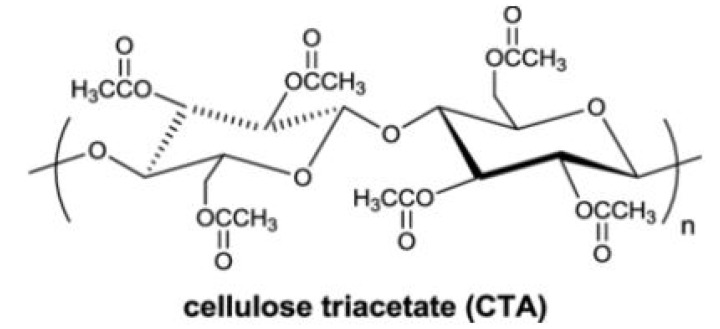
Molecular structure of CTA polymer unit.

CTA is a semi-crystalline polymer material. Due to high thermal stability and resistivity to most common solvents, CTA represents a suitable material for gas separation applications. Since the crystalline domains in polymers are generally not accessible to gas molecules, a decrease in gas solubility, diffusivity and permeability is common, especially for CO_2_ [26]. Moreover, CTA is a fascinating polymeric constituent for desalination due to its high salt rejection potential, practical mechanical strength, relatively low cost and ability to form a dense film [27]. The structure of CTA is shown in Figure 1. Modification of CTA can lead to a change in its crystallinity and, simultaneously, can increase polymer chain flexibility; both can increase gas permeability [28,29].

Herein, we report MMMs comprising CeO_2_ nanoparticles synthesized via a facile hydrothermal process and blended with CTA polymer. The membrane samples were prepared by a solution casting process, followed by the evaporation of the solvent at ambient temperature. The CTA-CeO_2_ matrix composition was selected based on the affinity of CeO_2_ towards CO_2_ [30] and the high thermal and mechanical stability of CTA. Similarly, because of the polar nature of the CTA polymer due to its numerous acetyl groups, it can also undergo highly oriented bonding and facile formation of a thin-film polymer by simple dissolution in an organic solvent. The synthesized MMMs were then used to evaluate the CO_2_ separation efficiency. The impact of the CeO_2_ loading on the physico-chemical, morphological and gas transport properties was studied. The gas permeation properties of the as-prepared membranes were evaluated using different gases based on CO_2_ capture from flue gas or syngas.

## 2. Materials and Methods

### 2.1. Chemicals

CTA (acetyl content 43–44%) was obtained from Acros Organics (Carlsbad, CA, USA). Cerium nitrate hexahydrated (Ce(NO_3_)_3_ 6H_2_O, 99.99%), sodium hydroxide (NaOH, 98.99%) and 1-methyl-2-pyrrolidinone (NMP, ACS reagent > 99.0%) were purchased from Sigma-Aldrich (Burlington, MA, USA). All the chemicals were used as received without any further purification.

### 2.2. Preparation of CeO_2_ Nanoparticles

First, 0.01 M Ce(NO_3_)_3_ 6H_2_O was dissolved in 100 mL of distilled water. To the solution, 5 M NaOH was added dropwise with constant stirring until complete precipitation occurred. After 4 h of continuous stirring at 400 rpm, the precipitate was transferred to an autoclave and subjected to hydrothermal treatment at 110 °C for 24 h. The precipitate was then washed, centrifuged 3–4 times using water/ethanol solvent, dried in the oven at 80 °C and finally annealed at 500 °C for 3 h.

### 2.3. Preparation of the CTA-CeO_2_ Mixed-Matrix Membrane

The calculated amount of CeO_2_ nanoparticles was mixed with the desired volume of solvent (NMP). First, the mixture was sonicated for 15 min (VWR^®^ Ultrasonic cleaner, USC-THD, Leicestershire, UK) and stirred for 3 h to ensure the homogeneous dispersion of nanoparticles. Next, the calculated amount of CTA (by weight of solution) was added to the initial mixture, dissolved in the NMP solvent and stirred for 18 h to obtain the optimal dispersion of nanoparticles in the polymer solution. The mixture was further sonicated for 10 min. Afterwards, the mixture was left unstirred for 4 h. The composition of all precursors used for the synthesis is listed in Table 1.

An average membrane thickness of 60 ± 5 µm was measured using a screw gauge fitted in an applicator (Elcometer 3580, Ulmer, Germany). The casted film was left in the air at ambient conditions to ensure complete solvent evaporation. Finally, MMMs containing the desired concentration of CeO_2_ were formed and used for further analysis. Membranes with 0, 0.32, 0.64 and 0.9 wt.% CeO_2_ were prepared and are referred to as CTA, CTA-CeO_2_ (0.32), CTA-CeO_2_ (0.64) and CTA-CeO_2_ (0.9), respectively, throughout the manuscript.

### 2.4. Material Characterization

The morphology of prepared samples was determined using scanning electron microscopy (SEM, Tescan LYRA, Brno, Czech Republic, 15 kV accelerating voltage, SE detector) equipped with energy dispersive spectroscopy (EDS, Oxford Aztec, 80 mm^2^, High Wycombe, UK) for the detailed analysis of element distributions within the materials and/or chemical microanalysis of elements present. Membranes were placed on double-sided adhesive tape made of carbon and covered by 2 nm of gold to ensure their high conductivity. Similarly, transmission electron microscopy (TEM) analysis of the nanoparticles was performed on a JEM-2200FS (Jeol, Japan) instrument, maintaining an accelerating voltage of 200 kV in TEM imaging mode. X-ray photoelectron spectroscopy (XPS) analysis was carried out in Omicron Nanotechnology and the primary X-ray beam was monochrome radiation of an Al lamp with energy of 1486.7 eV. Constant analyzer energy (CAE) mode was used, intensity calibration was performed based on previous measurements of copper and calibration constants were derived from the copper spectra. Measured spectra were evaluated by CasaXPS software, where, after intensity calibration, the areas of peaks and relative sensitivity factors (RSFs) from the database were used to determine concentrations. The known binding energy of the known element (C1s with binding energy of 284.8 eV) was used during the evaluation to calibrate the binding energy axis. The Brunauer Emmett-Teller (BET) surface area of CeO_2_ nanoparticles was determined at liquid N_2_ (77 K) temperature using the 3Flex (Micromeritics 3Flex, Heidelberg, Germany) adsorption analyzer. The 3D non-contact optical surface profiler NewView 9000 (ZYGO, Middlefield, CT, USA) was used for the non-contact surface topography analysis. Powdered X-ray diffraction (XRD) measurement was performed at a temperature of 273.5 K using a 2nd-Generation D2 Phaser X-ray diffractometer (Bruker, Bremen Germany) with Cu Kα radiation (λ = 0.15418 nm), SSD (1D mode) detector, coupled 2θ/θ scan type and continuous PSD fast scan mode. The range of measured Bragg angles was 5–80°. A voltage of 30 kV and a current of 10 mA were used. Fourier-transform infrared spectroscopy (FTIR) measurements were performed using a NicoletTM iS50 FTIR Spectrometer (Thermo Fischer Scientific USA) in absorbance mode. The spectra were taken in the wavenumber range of 400–3500 cm^−1^. Dynamic mechanical analysis (DMA) was carried out using DMA850 (TA Instruments, New Castle, DE, USA) in tensile mode to examine the glass transition temperature of MMMs and their mechanical properties. For each measurement, a sample gauge length of 10 mm and width of 4 mm were used. A start temperature of 100 °C, soak time of 5 min, end temperature setting of 300 °C (measurement auto-stop at specimen break), heating rate of 2 °C min^−1^, force amplitude of 0.02 N and pre-tension of 0.25 N were used.

Moreover, tensile stress–strain curves were measured using an Instron Universal Testing Machine 3365 (Instron, Norwood, MA, USA) equipped with pneumatic grips, rubber-coated sample gauge length (initial sample length between the grips) of 10 mm, sample width of 4 mm (on average) and a crosshead speed of 5 mm min^−1^ until specimen break. The measurement was carried out at ambient temperature. The thermal degradation of synthesized MMMs was measured by Setsys Evolution, Setaram (Caluire, France). The samples were heated in an aluminum crucible from 40 to 800 °C at a rate of 10 °C min^−1^ under a N_2_ atmosphere with a flow rate of 60 mL min^−1^. Similarly, to evaluate byproducts, TG analysis was carried out using a Pyris (Perkin Elmer, Waltham, MA, USA) coupled with FTIR (Perkin Elmer, Waltham, MA, USA). Differential scanning calorimetry (DSC) of the samples was measured by Setsys Evolution, Setaram (Caluire, France), using similar conditions to those of TG analysis. Samples were analyzed over a temperature range from ambient to 325 °C. The measurement was performed using a technique called STA, which is equal to the difference between TGA and DTA.

### 2.5. Gas Sorption and Permeation Measurements

CO_2_ and CH_4_ sorption experiments were performed gravimetrically at 25 °C in a pressure range from 0.1 to 1.5 MPa using a self-developed sorption apparatus equipped with a calibrated (McBrain) quartz spiral balance. A detailed description of the apparatus and the experimental procedure can be found elsewhere [31,32]. The gas permeation properties of all synthesized membranes were determined by O_2_, N_2_, H_2_, CO_2_ and CH_4_ in a custom-built time lag permeation setup. The membrane for gas permeation measurement was cut and tightly enclosed in a circular membrane permeation cell with an effective membrane surface area of about 2.14 × 10^−4^ m^2^. Prior to the gas sorption and permeation experiment, the membrane was kept in a vacuum oven overnight at 60 °C to ensure the removal of all trapped solvent. Similarly, the permeation cell was made air-free by applying a vacuum at both ends. In addition, after each gas experiment, the permeation cell was continuously evacuated with a vacuum to remove the gases present therein. The synthesized membranes were subjected to test gases and the data were collected using the SWeTr version 1.13 (2003, Neovision) data acquisition software. All permeation data were obtained when the steady state was reached. Each gas was run three times and the average value was recorded to minimize the experimental error.

The entire permeation curve was determined, including the initial transient, to allow the determination of the diffusion coefficients of the penetrants by the time lag method and the permeability coefficient from the steady-state pressure increase rate. The increase in the pressure in the fixed permeate volume was monitored as a function of time as soon as the membrane was exposed to feed gas at a pressure of 1.5 bar. For the given setup, the transient permeation curve, describing the pressure increase on the permeate side after exposure of the membrane to the feed gas, takes the following form [33,34].

(1) $$P_{t}=P_{0}+{\left(\frac{d_{p}}{d_{t}}\right)}_{0}+\frac{RTAl}{V_{p}\;V_{m}}p_{f\;}S\left(\frac{D_{t}}{t^{2}\;}-\frac{1}{6}-\frac{2}{\pi^{2}\;}\sum_{1}^{\infty }\frac{{\left(-1\right)}^{n}}{n^{2}}\;exp\left(-\frac{Dn^{2\;}\pi^{2\;}t\;}{t^{2}}\right)\right)$$

where *P_t_* is the permeate pressure (bar) at time t(s); *P*_0_ is the initial pressure, which is usually less than 0.05 mbar; (*d_p_/d_t_*)_0_ is the baseline slope, which is normally negligible for a defect-free membrane; *R* is the universal gas constant (8.314 × 10^−5^ m^3^ barmol^−1^ K^−1^); *T* is the absolute temperature (K); *A* is the active membrane area (m^2^); *V_p_* is the permeate volume (m^3^); *V_m_* is the molar volume of a gas at standard temperature and pressure (22.4 × 10^−3^ m^3^ STPmol^−1^ at 0 °C and 1 atm); *p_f_* is the feed pressure (bar); *S* is the gas solubility (m^3^ STPm^−3^ bar^−1^); *D* is the diffusion coefficient; *l* is the membrane thickness.

In the steady-state permeation condition, the exponential term approaches zero; hence, the equation becomes:

(2) $$P_{t}=P_{0}+{\left(\frac{d_{p}}{d_{t}}\right)}_{0}t+\frac{RTA}{V_{p}V_{m}}\times \frac{p_{f}P}{l}\left(t-\frac{l^{2}}{6D}\right)$$

A plot of *P_t_* versus t, after a long time, produces a straight line, which, upon extrapolation, intersects the time axis at $t=\frac{l^{2}}{6D}$, which describes the time lag (Ѳ󠆆) in permeation. These equations thus allow for the calculation of diffusion and permeability coefficients. Assuming the validity of the solution-diffusion model, the solubility coefficient was then determined indirectly by a simple relation: P=S×D. Generally, the gas permeability (*P*, 1 Barrer = 10^−10^ cm^3^ (STP) cm cm^−2^ s^−1^cm Hg^−1^) is defined by the following equation:

(3) $$P=\frac{lQ_{i}}{A\Delta P_{i}}$$

where *l* refers to the thickness of the membrane (µm), *Q* represents the volume flow rate (cm^3^ s^−1^, STP) of gas *i*, *A* is the effective membrane area (cm^2^) and ∆*P^i^* is the partial pressure difference across the membrane (cm Hg).

Similarly, the selectivity is defined by the expression:

(4) $$\alpha_{ij}=\frac{P_{i}}{P_{j}}$$

where $P_{i}$ and $P_{j}$ are the permeability of two pure gases ($P_{i}\;$> $P_{j}$), respectively.

## 3. Results and Discussion

### 3.1. Physico-Chemical Analysis

The effect of CeO_2_ addition on CTA membrane properties and gas separation behavior was determined. CTA-CeO_2_ asymmetric membranes prepared with different filler loadings (0.32, 0.64 and 0.9 wt.%) were characterized using the following techniques.

**Figure 2:**
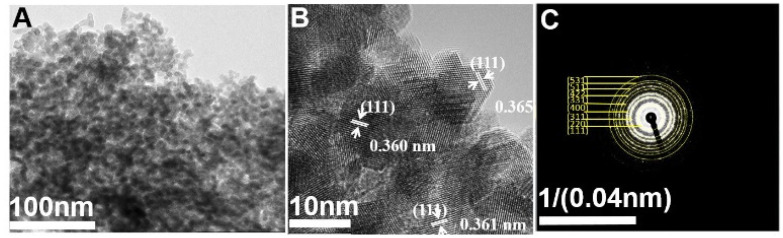
(**A**) TEM image, (**B**) HRTEM and (**C**) SAED pattern of CeO_2_ nanoparticles.

The TEM image reveals an average particle size of 10 ± 5 nm (Figure 2A). The *d*-spacing results obtained by HRTEM analysis are shown in Figure 2B. Lattice fringes of 0.362 ± 0.002 nm were determined, corresponding to the respective (111) plane of the cubic structure of CeO_2_. The distinguished fringes in SAED (Figure 2C) agree with the XRD results and suggest the well-defined crystal structures of CeO_2_ nanoparticles. Similarly, the chemical bonding nature of the CeO_2_ nanoparticles was analyzed using the XPS technique. The survey spectrum in Figure S1 in Supplementary Information (SI) indicates that the sample is mainly composed of Ce and O with binding energies of 880–910 eV (Ce3d) and 528–530 eV (O1s), respectively. High-resolution Ce3d spectra consist of several satellite peaks, which are assigned to the Ce^+4^ and Ce^+3^ oxidation states. The Ce^+3^ concentration can be correlated with oxygen vacancies on the CeO_2_ surface, which play roles in the catalytic activity of CeO_2_. The deconvoluted O1s spectra indicate the two principal binding energies associated with lattice oxygen (530 eV) and defect-oxide and/or hydroxyl-like groups (531.5–533 eV) [35]. The porosity of the synthesized nanoparticles was characterized by N_2_ adsorption-desorption analysis. The N_2_ adsorption-desorption isotherms show a characteristic hysteresis loop of type IV adsorption isotherms, indicating the mesoporous nature of the materials (Figure S2). The nanoparticles have a surface area of 88.31 m^2^ g^−1^ and the pore size is approximately 12 nm (11.64 nm in the adsorption section and 12.49 nm in the desorption section).

**Figure 3:**
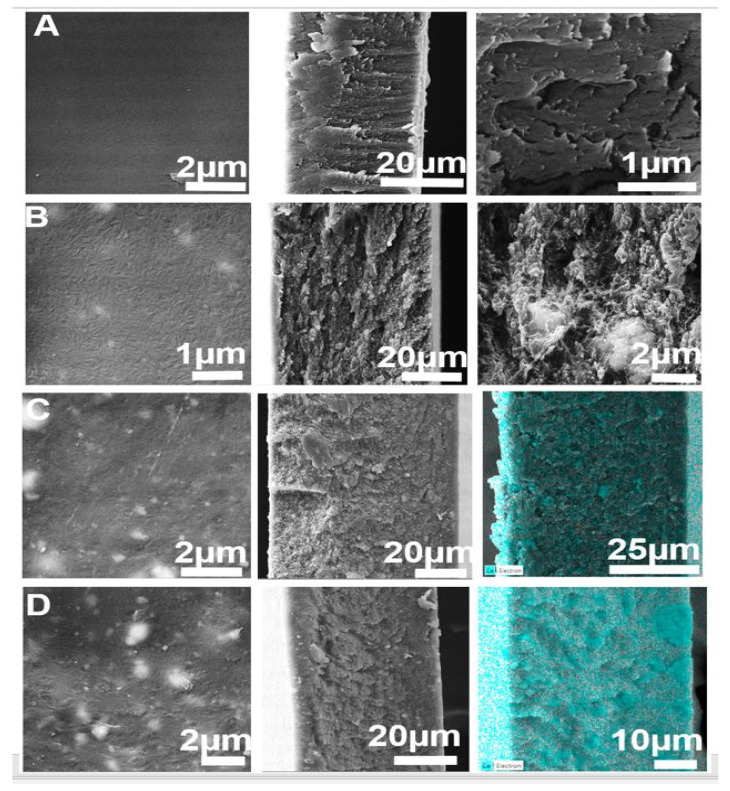
SEM images of the surface, cross-section, the magnified portion of the cross-section and EDS overlaid images: (**A**) CTA, (**B**) CTA-CeO_2_ (0.32), (**C**) CTA-CeO_2_ (0.64) and (**D**) CTA-CeO_2_ (0.9), across the rows, respectively.

The surface and the cross-sectional characteristics of the pristine and CeO_2_-blended CTA membranes were examined using the SEM technique with different magnifications and the results are presented in Figure 3. Pure CTA is observed to have a homogenous surface, while the bright spots in the MMM images (Figure 3B–D) represent the embedded CeO_2_ nanoparticles. As can be seen in the images, CeO_2_ nanoparticles are distributed over the CTA-CeO_2_ membrane surfaces with good dispersion. The cross-section SEM images confirm the dense character of the membranes. The EDS mapping of Ce over the cross-section of the CTA-CeO_2_ (0.64) sample in Figure S3D shows the homogeneous distribution of CeO_2_ nanoparticles throughout the membrane cross-section. The overlaid EDS images confirm the homogeneous distribution of the CeO_2_ nanoparticles throughout the membrane volume, suggesting that adequate sonication was implemented during dope solution preparation. The bulk/aggregated particles might have instantaneously broken and segregated into their smallest forms due to rigorous ultrasonication force, hence facilitating the dispersion of filler particles. This good distribution due to the high compatibility between the polymer and CeO_2_ nanoparticles was expected because of the favorable interaction between the acetate group (anionic) in the CTA polymer, which is capable of forming highly oriented hydrogen bonding and CeO_2_ via direct coordination to the central Ce metal ion or through the peripheral oxygen, forming a CTA-O-Ce hybrid [36,37]. Moreover, some aggregation of CeO_2_ nanoparticles after embedding can be observed for CTA with 0.9 wt.% loading. A highly magnified image of the cross-section of pristine CTA (Figure 3A) shows a dense morphology, while the MMMs possess voids (Figure S3A,B). A thick membrane was obtained due to delayed mixing in the phase separation with the evaporation of the solvent [38]. At the same time, voids were generated due to the interaction of the polymer and nanofillers. These free volume elements lead to the good permeability of gases.

**Figure 4:**
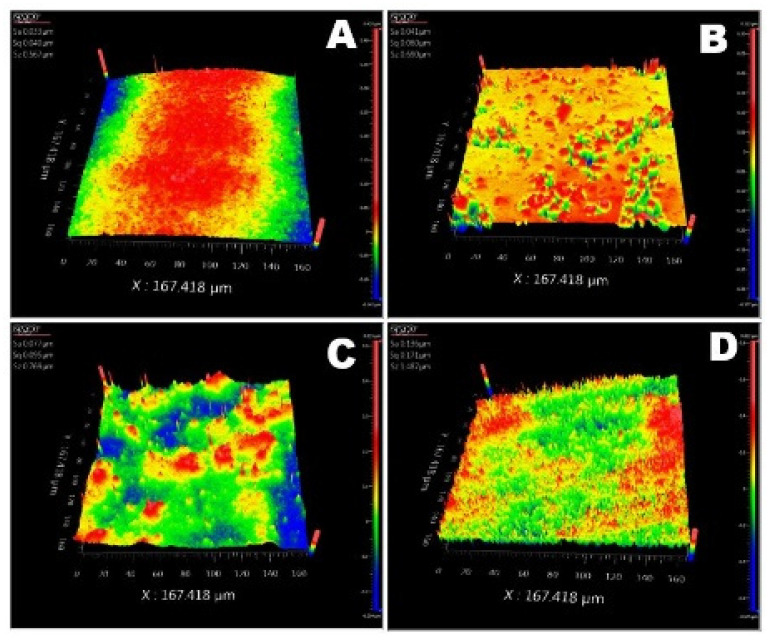
Surface topography analysis of the prepared pristine CTA and CTA with different concentrations of CeO_2_ nanoparticles: (**A**) CTA, (**B**) CTA-CeO_2_ (0.32), (**C**) CTA-CeO_2_ (0.64) and (**D**) CTA-CeO_2_ (0.9).

Figure 4 shows the surface roughness analysis results of the synthesized membranes. The composite membranes exhibit a rougher surface compared to the pristine membrane, i.e., higher root-mean-square height (Sq) (Table 2). Furthermore, the roughness increases with the increased concentration of nanoparticles in the blend materials. This increase in surface roughness is likely attributable to the partial aggregation of the nanoparticles.

**Figure 5:**
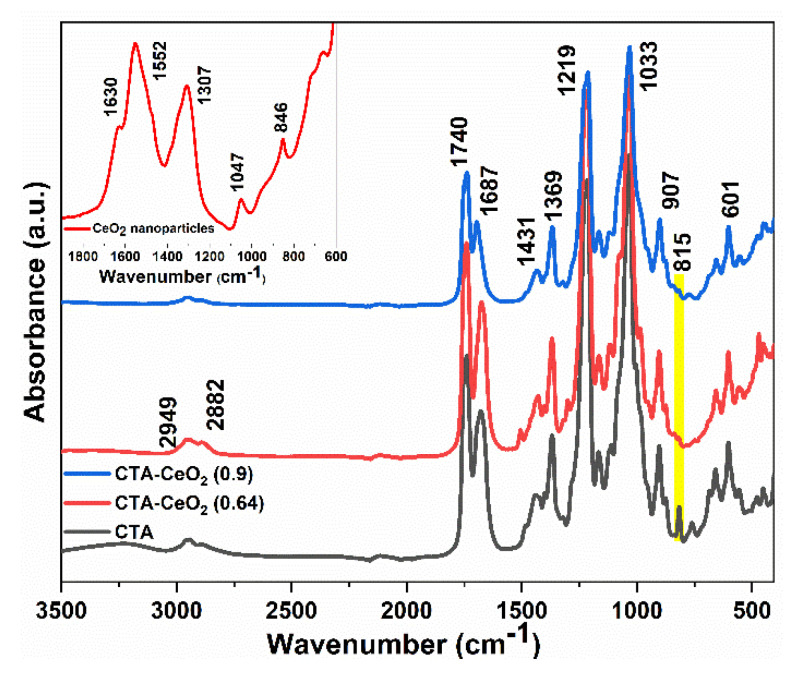
FTIR spectra of synthesized membranes with different concentrations of CeO_2_ nanoparticles.

The FTIR analysis (Figure 5) was carried out in the range of 3500–400 cm^−1^. The peaks at the wavenumbers 2949 and 2882 cm^−1^ correspond to C-H bonds. The peak at 1740 cm^−1^ corresponds to the stretching vibration of the C=O group. The band around 1687 cm^−1^ can be attributed to the COO band. The band at 1369 cm^−1^ represents the CH_3_ group for acetyl and the peak at around 1219 cm^−1^ corresponds to the C-O-C group of CTA [39]. The disappearance of the C-H deformation vibration at the wavenumber 815 cm^−1^ in the composite membrane can be attributed to the interaction of the CTA polymer with CeO_2_. The intensity of all characteristic peaks was lower in the blend membranes compared to the neat polymer. Similarly, the absorbance bands for CeO_2_ nanoparticles (inset) located at 1630, 1552 and 1307 cm^−1^ are attributed to the absorption of atmospheric CO_2_, indicating the high affinity of CeO_2_ nanoparticles towards CO_2_ absorption. The absorption bands below 900 cm^−1^ represent metal–oxygen stretching (Ce-O stretch), which confirms the formation of CeO_2_.

**Figure 6:**
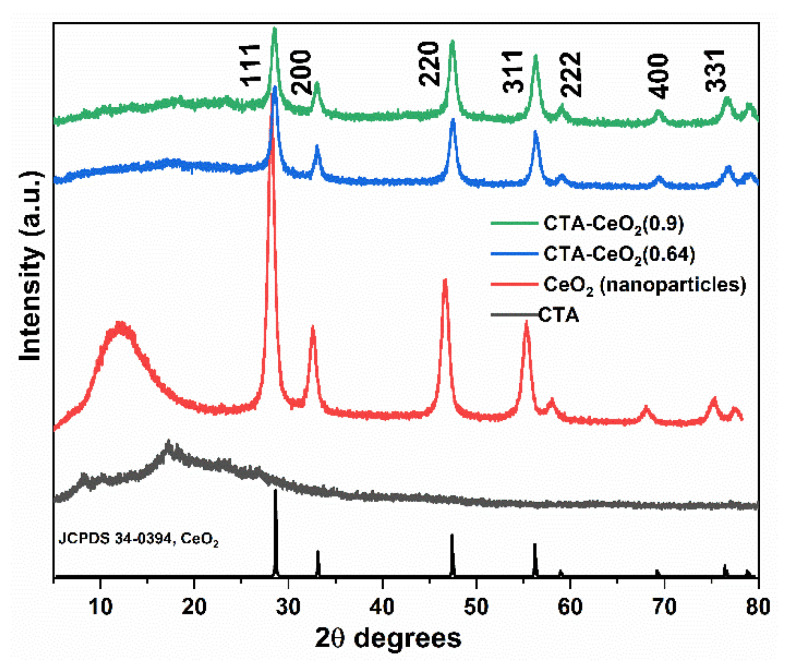
XRD spectral analysis of the synthesized CeO_2_ nanoparticles and CTA membrane with different concentrations of CeO_2_ nanoparticles.

Figure 6 shows the XRD patterns of the synthesized CeO_2_ nanoparticles, pristine CTA and MMMs. The XRD pattern of the pristine CTA film shows a broad diffuse peak centered at 19.5 2θ degrees, which is indicative of the semi-crystalline nature of CTA. Very sharp crystalline peaks of CeO_2_, corresponding to the standard JCPDS card No. 34-0394, were observed in the composite membrane. The peaks at 28.5°, 33.1°, 47.5°, 56.3°, 59.1°, 69.3° and 76.7° correspond to the (111), (200), (220), (311), (222), (400) and (331) planes of the fluorite structure of CeO_2_, respectively. The appearance of peaks of both CTA and CeO_2_ in the MMMs confirms the homogeneous blending of the CTA polymer matrix with CeO_2_ nanoparticles. The broad hump without sharp diffraction observed for the CTA peak at around 19.5 2θ degrees in the MMM spectra suggests an increase in the amorphous nature of the membranes. The diminished peak of CTA in MMMs could be attributed to CeO_2_ growing on the surface of CTA, which makes it harder to collect the diffraction data of CTA.

**Figure 7:**
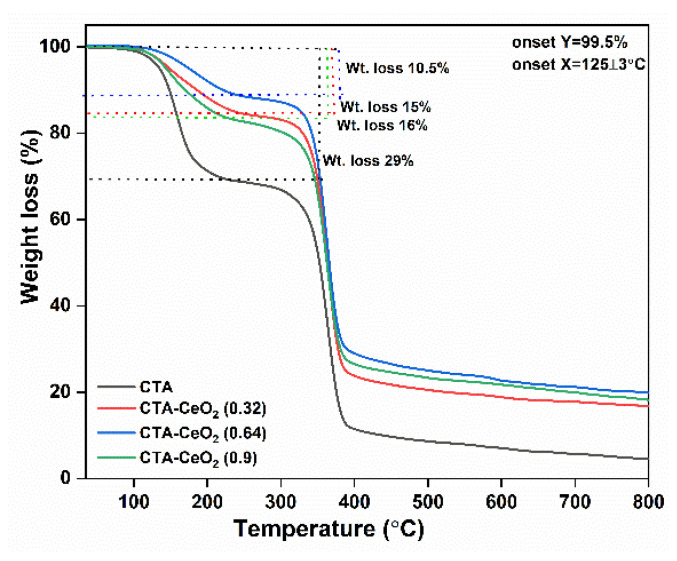
TGA thermogram of the pristine CTA and CTA-CeO_2_ MMMs.

The effects of the filler and its concentration on the thermal properties of pure CTA and modified CTA MMMs were examined by TGA. Generally, the addition of inorganic particles to the polymer tends to increase the thermal resistance of the membrane [40] and the same trend is observed for the prepared MMMs. The TGA (Figure 7) and its corresponding DTG (Figure S4) curves of pristine and CTA-CeO_2_ MMMs show two stages, from 120–190 and 310–390 °C, characterized by weight losses of 10–29% and 70–90%, respectively. The weight loss in the first phase can be attributed to water desorption, loss of entrapped solvent (as determined by FTIR measurement coupled with TGA (Figure S5) and decomposition of a small amount of diacetylated cellulose and some esterified chains [41]. The loss of the entrapped solvent led to substantial weight loss, which had a maximum rate at 150 °C and led to the loss of 29% of the initial mass. On the other hand, CTA without entrapped solvents has been reported to show no substantial weight loss before 190 °C [42]. The decrease in weight loss with the addition of CeO_2_ nanoparticles is attributed to the stabilization of the organic matrix because of the interaction with the CeO_2_ component [37]. The initiation of the more pronounced degradation process is observed at around 300 °C. The main decomposition step occurs in the range of 310–390 °C and leads to a weight loss of up to 70–90% for CTA-CeO_2_ and pristine CTA membranes, respectively. This sharp degradation is attributed to the removal of all acetate substituents and the formation of the pyranose ring in the polymer [43]. The thermal stability of the polymer increases with the addition of CeO_2_ nanoparticles, as demonstrated by the decrease in the weight loss from 29% to 10.5%. The residual mass of MMMs is high compared to the pristine membrane, which shows that the degradation temperature increases and weight loss decreases when incorporating CeO_2_ nanoparticles into the polymer matrix. This is presumably due to the good compatibility and strong interaction between nanoparticles and the polymer matrix. This compatibility between the polymer matrix and CeO_2_ nanoparticles is due to the strong coordination of Ce^4+^ with the acetate group of CTA. Therefore, interaction with nanoparticles likely reduces the mobility of CTA chains, thus slowing down the degradation process and decomposition occurs at a higher temperature [44].

Furthermore, to measure the thermal stability of the CeO_2_ nanoparticles only, TGA-DTG thermogram analysis was carried out (Figure S6). The results show a maximum weight loss of 3%, with major weight loss at around 40 °C. This is primarily due to the loss of water held on the surface by hydrogen bonding. The second minor weight loss peak at about 200 °C could be due to the loss of non-dissociative adsorbed water.

**Figure 8:**
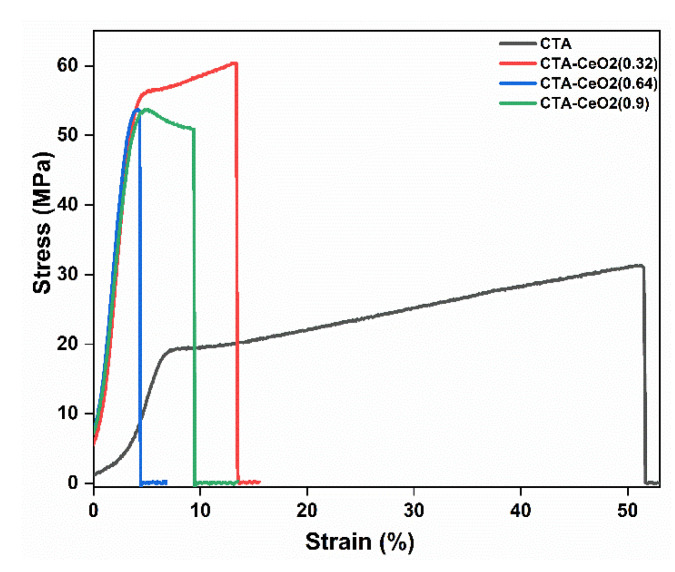
Tensile strength-elongation curves of the pristine and CeO_2_-modified CTA MMMs.

The effect of using CeO_2_ nanoparticles as fillers on the mechanical properties of the CTA MMMs depends on the polymer-particle interface properties. The mechanical tensile test results are shown in Figure 8 and Table 2. The pristine CTA exhibited a Young’s modulus of 1.24 GPa, an elongation strain of 51.29% and a tensile strength at break of 31.09 MPa. The addition of CeO_2_ to CTA MMMs increased both the tensile strength and Young’s modulus. With the incorporation of CeO_2_ nanoparticles, in contrast to pristine CTA properties, the strain drops unilaterally while the modulus increases to different extents, suggesting an efficient interphase load transport between phases during tension due to the high compatibility between the polymer and filler. The Young’s modulus of MMMs reaches a maximum at a filler loading of 0.64%. As the filler loading increases to 0.9%, the modulus of the membranes falls, probably due to poor compatibility between the CeO_2_ nanoparticle fillers and the CTA polymer matrix, leading to the failure of the transfer of the external force from the polymer matrix to the fillers. Similarly, at such a high filler concentration, some particles are likely to aggregate, resulting in decreasing compatibility between the CTA matrix and filler particles, which could reduce the modulus and the tensile strength of the MMMs from the discontinuous stress distribution during tension [40,45].

**Figure 9:**
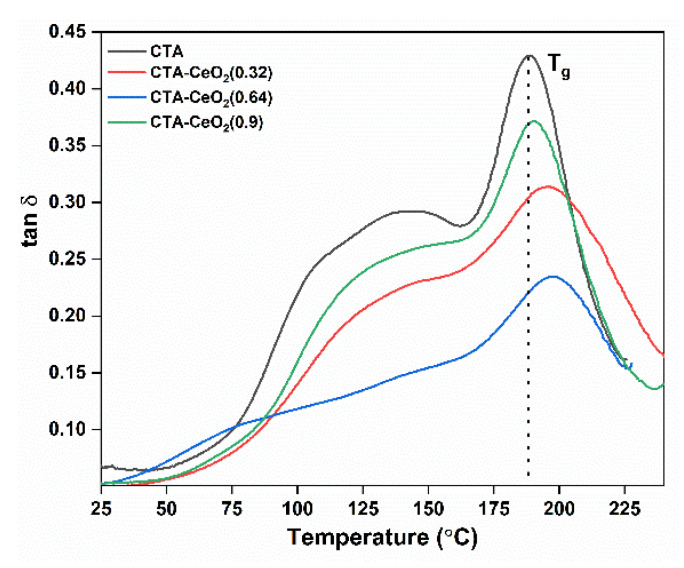
DMA plot showing the tan delta of pristine and CeO_2_-modified CTA membranes as a function of temperature.

Damping of the synthesized pristine CTA and MMMs is illustrated in Figure 9. Two peaks can be observed for every sample, with the major peak centered at 188.5 °C and a broad shoulder peak in between 100 and 175 °C. The shoulder peak is presumed to be caused by the relaxation between the crystalline and amorphous phases [46]. Tan delta peaks for the MMMs are broader than the pristine membrane, reaching the maximum for CTA-CeO_2_ (0.64 wt.%) samples and decreasing when the filler concentration is 0.9 wt.%. Similarly, the peak height for the major peak decreases with an increase in CeO_2_ filler concentration, reaches a minimum at 0.64 wt.% and then again increases at a CeO_2_ concentration of 0.9 wt.%. The lower peak height indicates good interfacial contact/adhesion [47]. The broadening of the peak shape and the decrease in the tan delta peak height could be attributed to the increase in amorphous characteristics of the MMMs because of the addition of CeO_2_ nanoparticles [48], which ultimately increases the polymer chain flexibility and improves the membrane permeability. Each sample shows a well-defined glass transition temperature (Tg) peak. The Tg values of MMMs are higher than those of the pristine membrane (Table 2) due to chain extension and rigidification [49].

The polymer crystallinity and melting behavior of the membranes can be determined with DSC by quantifying the heat associated with the melting (fusion) of the polymer. This heat is reported as percentage crystallinity by normalizing the observed heat of fusion to that of a 100% crystalline sample of the same polymer. In this study, samples were analyzed over a temperature range from ambient to 325 °C. A heating rate of 10° Cmin^−1^ was used with a N_2_ atmosphere around the sample.

**Figure 10:**
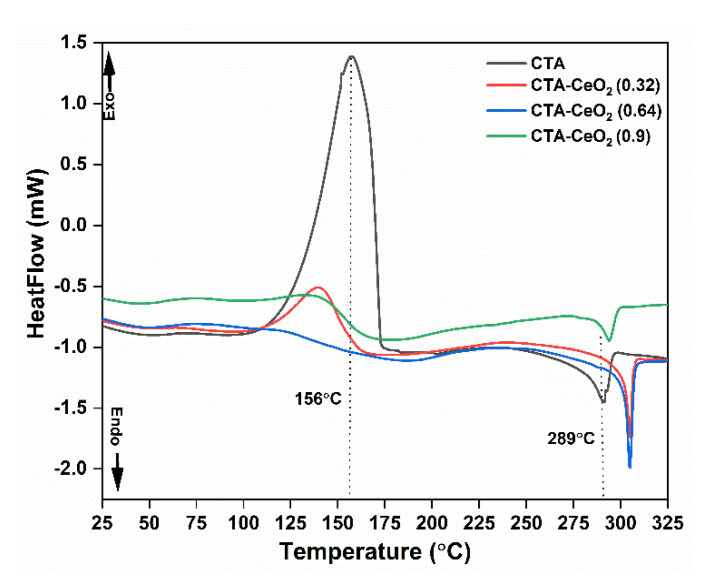
DSC thermogram of the pristine CTA and CTA-CeO_2_ MMMs.

Figure 10 shows the first heating DSC curve of the synthesized membranes. The results show one exothermic peak at around 156 °C and one endothermic peak at around 289 °C. The exothermic peak corresponds to crystallization, whereas the endothermic peak corresponds to fusion/melting. The DSC thermograms indicate that the incorporation of CeO_2_ in the polymer matrix has a direct influence on the melting temperature of CTA, increasing the melting temperature of the composites. The melting temperature of the composite appears at a higher temperature as compared to the pristine membrane, which is attributed to the good dispersion of CeO_2_ within the CTA matrix [50].

The degree of crystallinity was evaluated from the melting enthalpy in the first scan of each sample. The melting temperature was taken as the peak of the melting endotherm from the first scan of each membrane. The heat of fusion/melting was calculated from the integrated area under the melting peak. The enthalpy of fusion decreases with the addition of CeO_2_ nanoparticles: 7.31, 3.71, 4.53 and 2.05 Jg^−1^ for CTA, CTA-CeO_2_ (0.32), CTA-CeO_2_ (0.64) and CTA-CeO_2_ (0.9), respectively. This is indicative of a decrease in crystallinity. The degree of crystallinity (ℵ) of the modified membranes is calculated by the equation below:

(5) $$ℵ=\frac{\Delta H}{\Delta H^{{}^{\circ}}}\times 100\%$$

where ΔH (Jg^−1^) is the melting enthalpy of the synthesized membrane and $\Delta H^{{}^{\circ}}$ is the melting enthalpy for a 100% crystalline sample of pure CTA. The heat of fusion for a perfect CTA crystallite is taken to be 34.31 Jg^−1^ [26]. Assuming that this heat of fusion applies to all modified membranes, the crystallinity of the MMMs here decreases significantly as compared to pristine CTA: 21.31%, 10.81%, 13.21% and 5.97% for CTA, CTA-CeO_2_ (0.32), CTA-CeO_2_ (0.64) and CTA-CeO_2_ (0.9), respectively. This indicates the strong interaction between the polymer and fillers. This change in crystallinities should have a significant effect on the gas transport properties.

### 3.2. Gas Separation Performance of MMMs

**Figure 11:**
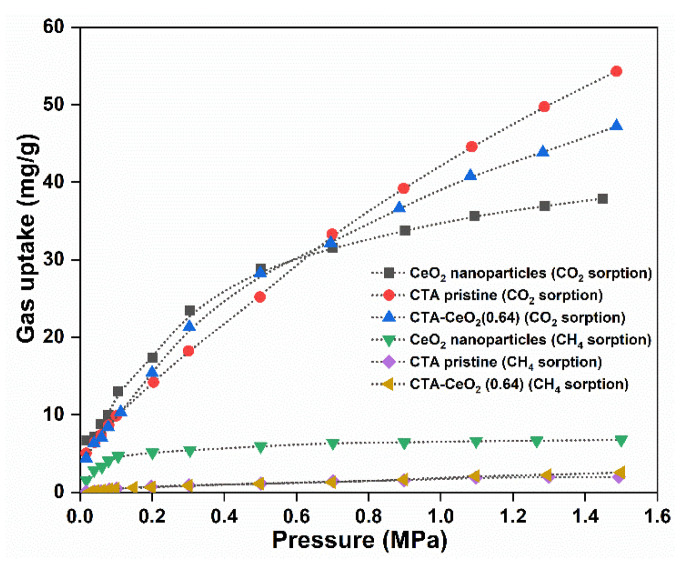
CO_2_ and CH_4_ gas sorption isotherms of CeO_2_ nanoparticles, pristine CTA and CTA-CeO_2_ MMMs.

CO_2_ and CH_4_ gas sorption isotherms of CeO_2_ nanoparticles, pristine CTA and CTA-CeO_2_ (0.64) membranes are shown in Figure 11. The sorption of CO_2_ is higher than that of CH_4_ for all sets of samples. This observation is related to the high condensability (high critical temperature) relative to CH_4_ and potentially to the specific interaction of CO_2_ molecules with CeO_2_ nanoparticles. CTA-CeO_2_ (0.64) MMMs show low CH_4_ uptake affinity, while CeO_2_ nanoparticles show comparably high CH_4_ sorption, already showing saturation at atmospheric pressure of CH_4_. The order of the CO_2_ sorption affinity of the samples in a pressure range from 0.02 to 0.8 MPa is CeO_2_ NP > CTA-CeO_2_ (0.64) > pristine CTA, while above this pressure range, the CO_2_ uptake affinity increases in the reverse order: CeO_2_ NP < CTA-CeO_2_ (0.64) < pristine CTA. This order is attributed to the saturation of the active sites of CeO_2_ with CO_2_ gas with an increase in CO_2_ concentration, leading to a decrease in CO_2_ uptake. This also decreases the CO_2_ uptake affinity of the CTA-CeO_2_ (0.64) sample. Moreover, at lower pressures, the CO_2_ uptake affinity of MMMs is higher than that of pristine CTA, which likely causes an increase in the permeability/selectivity of the gas pair.

The CO_2_ sorption isotherms of the samples were further predicted with the dual mode sorption (DMS) model [51].

(6) $$v=k_{D}p+\frac{c_{H}bp}{1+bp}$$

where 𝑣 is the sorbent concentration; p is the adsorbate pressure; $k_{D}$ is Henry’s law solubility constant; $c_{H}$ is the Langmuir sorption capacity constant, which characterizes the sorption capacity for a given penetrant in a low pressure region; b is the Langmuir affinity constant, which represents the ratio of the rate constant of sorption and desorption of the penetrants and, therefore, quantifies the tendency of a given penetrant to sorb according to Langmuir mode. The fitting parameters and corresponding coefficients of determination (R^2^) are listed in Table S1 and the fitted graph is shown in Figure S7.

The predicted value from the DMS model and the experimental data fit well for CeO_2_ nanoparticles and pristine CTA membranes; however, for the CTA-CeO_2_ MMMs, the predicted isotherms fit well with the experimental values at pressures below 0.8 MPa, while at higher pressures, the values deviate from the experimental results. This can be attributed to the adverse effect of the incorporation of CeO_2_ into CTA on the CO_2_ sorption affinity. A further study is essential. Furthermore, to confirm the reliability of the model for the given samples, we also used the Guggenheim–Anderson–De Boer (GAB) model [52]:

(7) $$v=\frac{v_{m\;}hp^{*}p}{\left(p^{*\;}-p\right)\left(hp+p^{*}-p\right)}$$

where v is the adsorbed mass of adsorbate per mass of pure adsorbent, $v_{m}$ is the capacity of the first adsorption monolayer, h represents the fold increase in the binding strength of adsorbate molecules in the first layer relative to the successive layers, *p* is adsorbate pressure and $p^{*}$ is a pressure-independent constant. The experimental and predicted adsorption isotherms of CO_2_ in pristine CTA, CeO_2_ nanoparticles and CTA-CeO_2_ MMMs are shown in Figure S8. The fitting parameters and corresponding coefficients of determination (R^2^) are listed in Table S1. Similar to the DMS model, the predicted isotherms based on the GAB model also fit well with the experimental data for CeO_2_ nanoparticles and pristine CTA membranes; however, for the CTA-CeO_2_ MMMs, the predicted isotherms deviate from the experimental values at pressures from 0.8 to 1.5 MPa.

The gas permeation properties of the CTA-CeO_2_ membranes, i.e., permeance and selectivity, were evaluated by using pure gases: O_2,_ N_2_, H_2_, CO_2_ and CH_4_. The separation performance of gases was evaluated at 25 °C with a transmembrane pressure of 1 torr and feed pressure of 1.5 bar. The values of the gas permeabilities are shown in Table 3. The gas permeability in both pristine and CTA-CeO_2_ generally decreases with an increase in the molecular size of the gas in the following order: H_2_ > CO_2_ > O_2_ > N_2_ > CH_4_. Pristine CTA is a barrier material for gases owing to its semi-crystalline nature. Even the fast-permeating species H_2_ has a permeability of 4.39 Barrer, which decreases to less than 1 Barrer for O_2_, N_2_ and CH_4_. The addition of a low amount of CeO_2_ causes an increase in permeability. The gas permeability increases as the concentration of CeO_2_ filler increases from 0 to 0.64 wt.%, accompanied by a decrease in permeability at 0.9 and 1.4 wt.%. The highest permeabilities for H_2_, CO_2_, O_2_, N_2_ and CH_4_ in this study were obtained for CTA-CeO_2_ (0.64): 15.09, 9.67, 9.07, 2.97 and 2.89 Barrer, respectively. The permeability results in Table 3 show that the presence of CeO_2_ particularly favors the transport of less permeable gases, such as N_2_ and CH_4_, as well as species with high solubility, such as CO_2_. The increases in the permeation rates of these species are greater than those of H_2_ and O_2_; hence, the selectivity of CO_2_/CH_4_ and CO_2_/N_2_ gas pairs decreases with the increase in the CeO_2_ concentration. The selectivity of H_2_/CO_2_ remains relatively constant. It should be noted that the gas permeation properties of the MMMs can be efficiently tailored, even with the incorporation of extremely low loadings of fillers. Compared to other fillers that require high loadings to enhance permeability or selectivity, using a low loading of fillers is advantageous given its potential to mitigate particle aggregation and ensure the uniform distribution of fillers in the polymer matrix. For all penetrant gases in this study, the permeability of the CTA-CeO_2_ membranes is higher than that of pristine CTA membranes and permeability increases with increasing CeO_2_ content. This result does not corroborate the conventional Maxwell model, which predicts that the gas permeability of an MMM containing nonporous filler is lower than that of the pristine polymer and decreases with increasing filler content due to a decrease in diffusivity because of the increased tortuosity in the polymer matrix [53]. Thus, it can be presumed that the gas transport properties of the CTA-CeO_2_ MMMs are associated with a substantial change in free volume due to the introduction of CeO_2_ nanoparticles. This increase in total volume due to the disruption of chain packing provides more spacious pathways, thus leading to an increase in diffusivity, thereby increasing permeability. An increase in total free volume results in increases in diffusion and solubility coefficients (as supported by diffusivity and solubility data), thus leading to an increase in permeability. Moreover, the permeability of large gas molecules is further enhanced by the addition of CeO_2_ due to the increase in free volume, which increases the diffusion coefficient and permeability and results in a reduction in pure gas selectivity. The nonlinear increase in gas permeability with CeO_2_ content may be attributed to a tendency of free volume to increase due to nanoparticle agglomeration [54]. He et al. reported that the addition of nonporous fillers (silica) to glassy polymers with high free volume increased the total free volume of the membrane by disrupting the polymer chain packing [53]. They also reported the increase in the diffusion and solubility coefficients as a function of increasing filler content. Similarly, the permeability of the synthesized MMMs depends on the crystallinity and the void volume fraction of the membrane. Thus, a decrease in the crystallinity of the CTA matrix, as illustrated by DSC and DMA and the formation of the higher void volume, as observed using SEM, increases permeability. Analogously, the selectivity of the synthesized MMMs changes in the reverse order with the increase in CeO_2_ concentration. It can be presumed that for the higher concentration of fillers in the polymer matrix (0.9% and 1.4%), the mixed-matrix tortuous pattern will increase. This will restrict the diffusion of larger molecules while allowing the diffusion of small molecules with less resistance, which in turn improves the gas selectivity [55].

**Figure 12:**
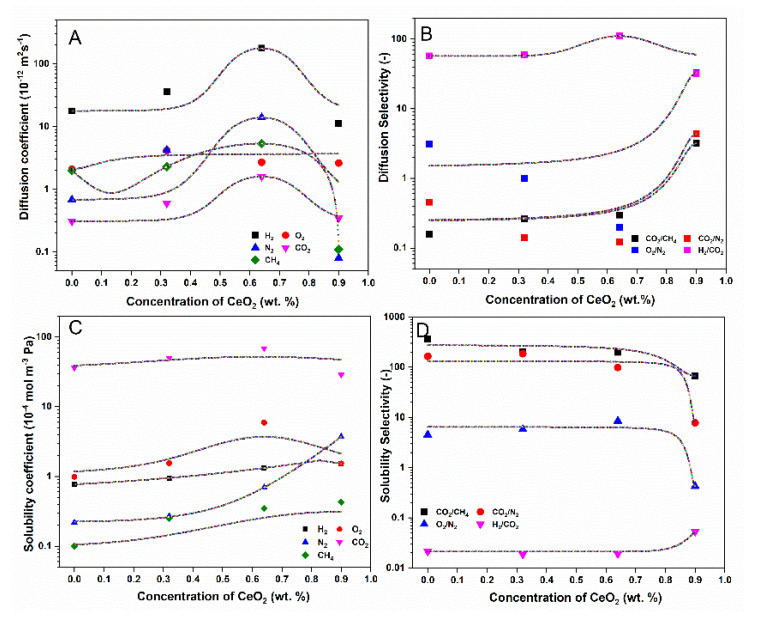
(**A**) Diffusion coefficient, (**B**) diffusion selectivity, (**C**) solubility coefficient and (**D**) solubility selectivity between selected gas pairs as a function of CeO_2_ filler concentration. Lines are plotted for visualization.

The performance of the different gas pairs was compared with the respective 2008 upper bound curve [4], as shown in Figure S9. It can be observed that the incorporation of CeO_2_ nanoparticles into the CTA polymeric matrix improves their performance. As shown in Figure 12, diffusion coefficient values for all gases in the study increase with an increase in CeO_2_ concentration, reaching a maximum at 0.64 wt.%. Upon further increase in CeO_2_ content (0.9 wt.%), the diffusion coefficient decreases, which is in line with the permeability results. A decrease in diffusion can reduce the free volume, chain mobility and interchain distance and increase the crystallinity of the hybrid membrane [56]. A higher concentration of CeO_2_ can increase the tortuosity of the diffusion path in MMMs, which results in a decrease in the diffusion coefficient. The solubility coefficient is much less dependent on the CeO_2_ concentration than the diffusion coefficient. The solubility of small gas molecules such as H_2_, CO_2_ and O_2_ increases up to a CeO_2_ concentration of 0.64 wt.% and then decreases at higher concentrations, whereas the solubility of larger gas molecules such as N_2_ and CH_4_ increases with the increase in the CeO_2_ concentration. However, the solubility of CO_2_ is higher than its diffusivity, whereas, for the rest of the gases in this study, the values are reversed, with higher diffusivity than solubility. The higher solubility of CO_2_ as compared to other gases is mainly attributed to the high affinity between this gas and the polymer matrix, which could be due to acid-base (Lewis) interactions (electron donor-acceptor) between CO_2_ and CeO_2_ [57] and the high condensability of CO_2_. At a higher concentration of CeO_2_ (0.9 wt.%), due to the aggregation of nanoparticles, the effective surface area on the nanoparticles decreases, consequently decreasing the active sites available for interaction with CO_2_ molecules. The increase in permeability due to the presence of CeO_2_ is thus mainly attributed to the synergistic effect of the solubility and diffusivity properties. Hence, gas transport through the synthesized MMMs is attributed to a solution-diffusion mechanism. The higher solubility of CO_2_ as compared to other gases in the study, combined with the increase in the diffusion coefficient value with the increase in CeO_2_ concentration, makes the synthesized MMMs suitable to use in flue gas separation.

It is also worth mentioning that CTA, being a polar polymer with a number of acetyl groups that are capable of forming dipolar interactions with polarizable CO_2_ molecules, is prone to CO_2_ plasticization. Bos et al. reported that the CO_2_-induced plasticization pressure of CTA is 10 bar of feed pressure [58]. In the current work, the gas permeation experiments were carried out at a constant feed pressure of 1.5 bar and we presume that the probability of plasticization at this low pressure is negligible.

The selectivity of CO_2_/CH_4_ and CO_2_/N_2_ is higher in comparison to the O_2_/N_2_ gas pair. The selectivity follows the order CO_2_/CH_4_ > CO_2_/N_2_ > O_2_/N_2_ > H_2_/ CO_2_. Due to the higher affinity of CO_2_ to CeO_2_ [57], it is more soluble in membranes, resulting in higher selectivity than O_2_/N_2_. Moreover, the selectivity of CO_2_/N_2_ is greater than that of CO_2_/CH_4_, despite the larger kinetic size of CH_4_ (3.8 Å) as compared to N_2_ (3.64 Å). This could be because the solubility of N_2_ is higher than that of CH_4_ at the given membrane matrix concentration. The lower value of the gas selectivity of O_2_/N_2_ could be due to the similar condensability of the two gases, similar gas kinetic diameters (O_2_: 3.46 Å; N_2_: 3.64 Å) and lack of interaction with the membrane or CeO_2_ nanoparticles. Similarly, the addition of CeO_2_ nanoparticles to the CTA membrane positively affects the H_2_ and CO_2_ permeability. However, the selectivity of H_2_/CO_2_ was not improved because the higher diffusion rate of H_2_ and higher solubility rate of CO_2_ increased the permeabilities of both gases in the same proportion, thus resulting in almost the same selectivity value. The solubility selectivity of CO_2_/CH_4_ is larger than that of other gas pairs in the study. All membrane samples with up to 0.64 wt.% CeO_2_ maintained almost the same solubility selectivity value as that of the pristine CTA membrane filler concentration and then it decreased at the higher concentration of 0.9 wt.% CeO_2_. A similar trend is observed for CO_2_/N_2_, while for H_2_/CO_2_, the solubility selectivity increases at higher concentrations. Similarly, the diffusion selectivity of all gas pairs in the study increases with the increase in CeO_2_ concentration in the membrane matrix. The diffusion selectivity of the CO_2_/CH_4_ gas pair is lower than that of the other gas pairs in the study. The diffusion selectivity of CO_2_/CH_4_ and H_2_/CO_2_ increases with the increase in CeO_2_ concentration in the membrane matrix, while the other two gas pairs show the reverse trend. Hence, the gas permeabilities and selectivities are strongly related to the CeO_2_ concentration, indicating the significant role of CeO_2_ fillers in MMMs for gas separation. According to the literature, CeO_2_ is widely used as a catalyst for the conversion of CO_2_ to methanol and other valuable fuels. The relative ease of switching between Ce^+4^/Ce^+3^ oxidation states and high oxygen storage capacity are considered key factors for the catalytic activity of CeO_2_ [59]. These properties can be adversely impacted by the presence of acid gases (CO_2_, SO_2_, etc.), even at low concentrations. Senanayake and Mullins observed that CO_2_ interacts with both oxidized and reduced CeO_2_ [60]. It was found that the CO_2_ adsorbed on the CeO_2_ surface in the form of bridged, monodentate and bidentate carbonates and bicarbonates [61]. Thus, considering the high thermal and mechanical properties and the high affinity of CeO_2_ towards CO_2_, the synthesized MMMs, with optimized gas permeation parameters, are believed to be a promising membrane matrix for flue and/or biogas treatment.

## 4. Conclusions

In conclusion, we prepared a series of MMMs by blending CeO_2_ with CTA polymer, followed by the facile solution-casting method. The physico-chemical, thermal and mechanical properties of the synthesized membranes were analyzed. The blended nanoparticles exhibited high compatibility with the CTA polymer. The results show homogeneous CeO_2_ distribution throughout the membrane and minimum agglomeration up to a concentration of 0.64 wt.%. The MMMs possessed better thermal and mechanical stability as compared to the pristine membrane. The membrane matrix with 0.64 wt.% CeO_2_ showed higher gas sorption affinity for CO_2_ at pressures below 0.8 MPa, as well as permeation for all of the single gases studied (O_2,_ N_2_, H_2_, CO_2_ and CH_4_). Moreover, the greatest increase in permeability (9.09 Barrer) was obtained for CO_2_. The high affinity of MMMs towards CO_2_ gas permeation compared to other low molecular weight gases lays the foundation for its application in flue gas or biogas treatment. Owing to the facile fabrication technique and effective improvement in the mechanical and thermal properties of the prepared MMMs, the current gas separation results demonstrate the potential of these materials for the gas separation process. However, there is still much room for separation performance improvement, e.g., by optimizing the permeation parameters.

## Figures and Tables

**Table 1:** Composition of polymer, nanoparticles and solvent used in MMM preparation.

#	Sample	wt.% of CTA	wt.% of CeO_2_	wt.% of NMP
1	CTA	5.75	0.0	94.25
2	CTA-CeO_2_ (0.32)	5.75	0.32	94.93
3	CTA-CeO_2_ (0.64)	5.75	0.64	93.61
4	CTA-CeO_2_ (0.9)	5.75	0.9	93.35

**Table 2:** Glass transition temperature, mechanical properties and surface roughness comparison of the synthesized membranes.

Sample Code	Glass Transition Tempt (Tg) (°C)	Mechanical Properties (25 °C)			Surface Roughness		
		Young’s Modulus (GPa)	Elongation Strain at Break (%)	Tensile Strength (MPa)	Arithmetic Mean Height Sa (µm)	Root-Mean-Square Height Sq (µm)	Maximum Height Sz (µm)
CTA	188.5	1.24	51.29	31.09	0.033	0.040	0.57
CTA-CeO_2_ (0.32)	194.1	1.39	13.31	60.38	0.041	0.060	0.70
CTA-CeO_2_ (0.64)	197.3	1.62	4.71	53.75	0.077	0.095	0.77
CTA-CeO_2_ (0.9)	190.5	1.47	4.18	53.74	0.136	0.171	1.49

**Table 3:** Permeability of the synthesized MMMs and their respective selectivity for gases at a temperature of 25 °C and pressure of 1 torr.

Sample	Permeability (Barrer)					Selectivity			
	H_2_	O_2_	N_2_	CO_2_	CH_4_	CO_2_/CH_4_	CO_2_/N_2_	O_2_/N_2_	H_2_/CO_2_
CTA	4.39	0.65	0.03	3.01	0.12	25.08	99.0	21.67	1.48
CTA-CeO_2_ (0.32)	7.2	0.68	0.35	6.89	0.28	24.61	19.69	1.94	1.04
CTA-CeO_2_ (0.64)	15.09	9.07	2.97	9.67	2.89	3.34	3.26	3.05	1.56
CTA-CeO_2_ (0.9)	5.19	0.43	0.02	2.66	0.03	88.67	133.0	21.50	1.95
CTA-CeO_2_ (1.4) *	4.9	0.89	0.04	3.56	0.18	19.78	89.0	21.25	1.38

* This membrane was prepared only for gas permeability experiments to confirm the hypothesis related to the effect of CeO_2_ content.

## Supplementary Materials

The following are available online at https://www.mdpi.com/article/10.3390/membranes11080632/s1, Figure S1. XPS spectrum of the CeO_2_ nanoparticles: (A) survey spectrum, (B) Ce3d, and (C) O1s. Figure S2 BET surface area of CeO_2_ nanoparticles. Figure S3 SEM images of the magnified portion of the cross-section of; A) CTA-CeO_2_ (0.64): B) CTA-CeO_2_ (0.9) showing the formation of the voids: C) portion of cross-section of CTA-CeO_2_ (0.64) for EDS mapping: and D) Element Ce mapping over the cross-section of image in C showing the homogeneous distribution of Ce. Figure S4. DTG plot for pristine and CeO_2_-loaded CTA membranes. Figure S5. FTIR spectra of the product from samples measured during TGA measurement: (A) CTA, (B) CTA-CeO_2_ (0.64). Figure S6. TGA-DTG thermogram for CeO_2_ nanoparticles. Figure S7. Sorption isotherms of CO_2_ in pristine and CTA–CeO_2_ MMMs (filled symbol indicates the experimental data, whereas the dashed/solid line indicates the value predicted using the DMS model). Figure S8. Sorption isotherms of CO_2_ in pristine and CTA–CeO_2_ MMMs (filled symbol indicates the experimental data, whereas the dashed/solid line indicates the value predicted using the GAB model). Figure S9. Robeson upper bound comparison for the different gas pairs in this study. The number in each symbol indicate the MMM samples: 1) CTA, 2) CTA-CeO_2_ (0.32), 3) CTA-CeO_2_ (0.64), and 4) CTA-CeO_2_ (0.9). Table S1. Fitting parameters for DMS and GAB models for CO_2_ gas sorption.
