# Supplementary material for: CeO2-Blended Cellulose Triacetate Mixed-Matrix Membranes for Selective CO2 Separation
Source: Membranes (Basel). 2021 Aug 17;11(8):632. doi: 10.3390/membranes11080632 (PMC8400081; doi:10.3390/membranes11080632)
Supplement: Supplementary file 1 [file membranes-11-00632-s001.zip › membranes-1305626-supplementary.pdf]

membranes

Supplementary Information

CeO_2-Blended Cellulose Triacetate Mixed-Matrix Membranes for Selective CO_2 Separation

Chhabilal Regmi [a,*], Saeed Ashtiani [a], Zdeněk Sofer [b], Zdeněk Hrdlička [c], Filip Průša [d], Ondřej Vopička [a] and Karel Friess [a,*]

[a] Department of Physical Chemistry, University of Chemistry and Technology, Technická 5, 16628 Prague, Czech Republic
[b] Department of Inorganic Chemistry, University of Chemistry and Technology, Technická 5, 16628 Prague, Czech Republic
[c] Department of Polymers, University of Chemistry and Technology, Technická 5, 16628 Prague, Czech Republic Department of
[d] Metals and Corrosion Engineering, University of Chemistry and Technology, Technická 5, 16628 Prague, Czech Republic
* Correspondence: regmic@vscht.cz; friessk@vscht.cz

1. Results

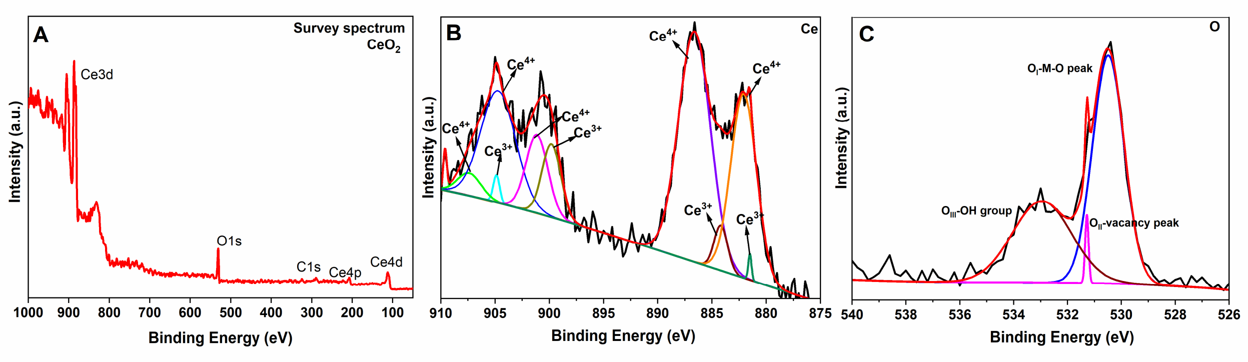

Figure S1. XPS spectrum of the CeO_2 nanoparticles: (A) survey spectrum, (B) Ce3d, and (C) O1s.

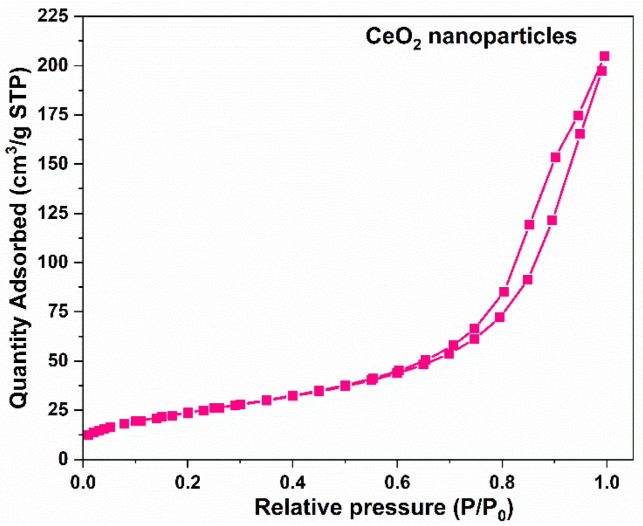

Figure S2 BET surface area of CeO_2 nanoparticles.

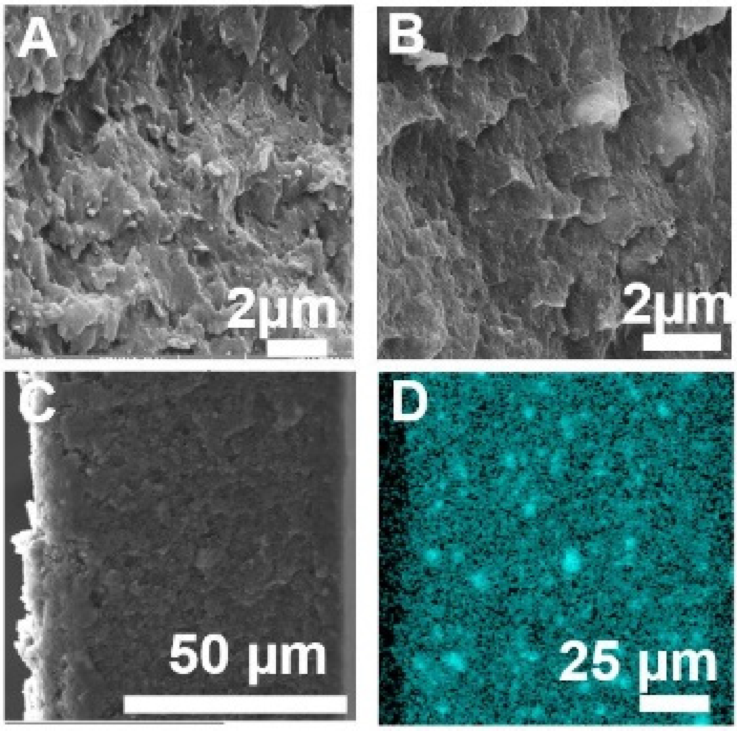

Figure S3 SEM images of the magnified portion of the cross-section of; A) CTA-CeO_2 (0.64) : B) CTA-CeO_2 (0.9) showing the formation of the voids : C) portion of cross-section of CTA-CeO_2 (0.64) for EDS mapping: and D)Element Ce mapping over the cross-section of image in C showing the homogeneous distribution of Ce.

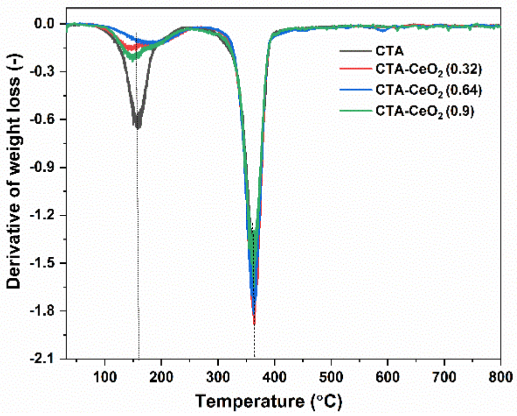

Figure S4. DTG plot for pristine and CeO_2-loaded CTA membranes.

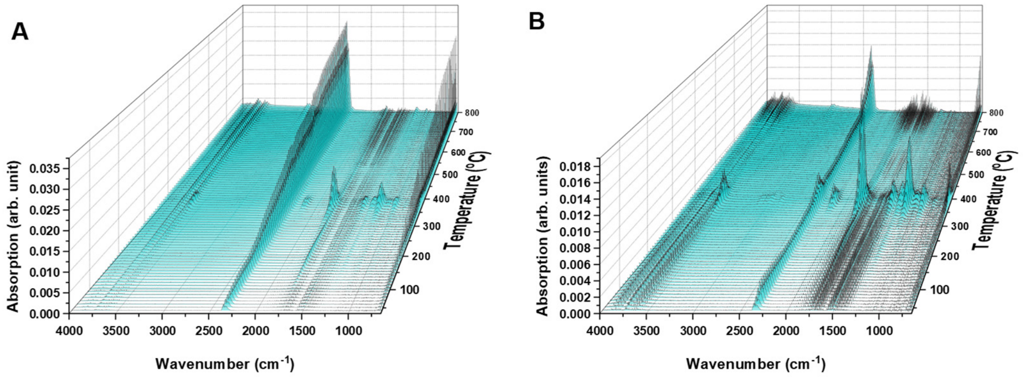

Figure S5. FTIR spectra of the product from samples measured during TGA measurement: (A) CTA, (B) CTA-CeO_2 (0.64).

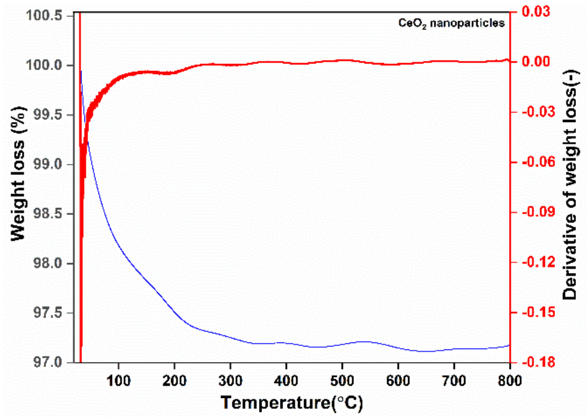

Figure S6. TGA-DTG thermogram for CeO_2 nanoparticles.

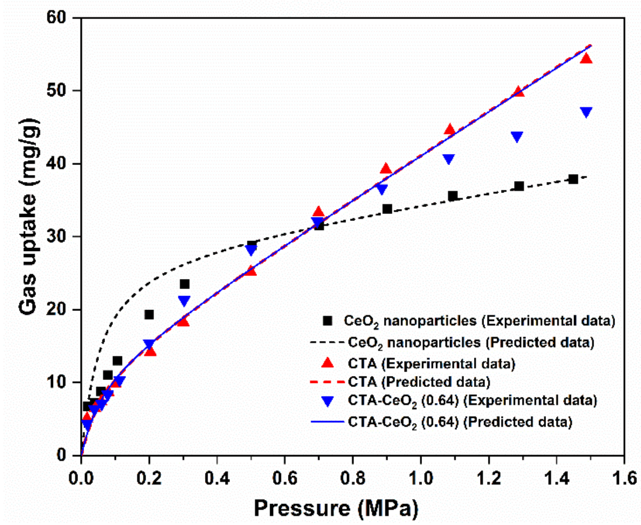

Figure S7. Sorption isotherms of CO_2 in pristine and CTA–CeO_2 MMMs (filled symbol indicates the experimental data, whereas the dashed/solid line indicates the value predicted using the DMS model).

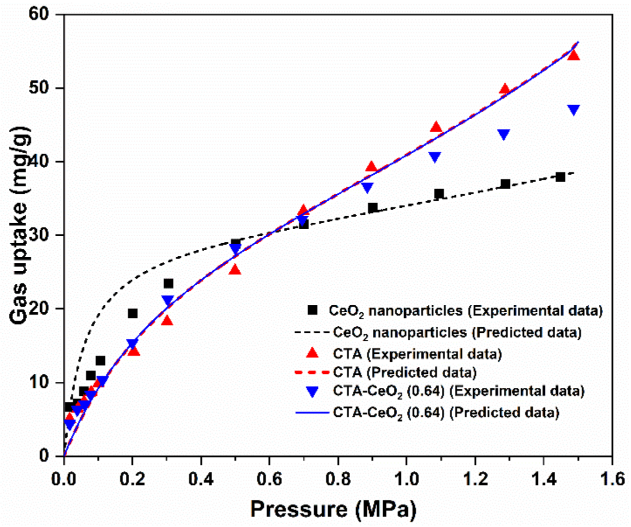

Figure S8. Sorption isotherms of CO_2 in pristine and CTA–CeO_2 MMMs (filled symbol indicates the experimental data, whereas the dashed/solid line indicates the value predicted using the GAB model).

Table S1. Fitting parameters for DMS and GAB models for CO_2 gas sorption.

Samples	Fitting Parameters (DMS Model)				Fitting Parameters (GAB Model)			
	k_D	C_H	b	R^2	v_m	h	P*	R^2
CTA	29.87	11.92	16.17	0.99	35.80	11.23	3.51	0.99
CeO_2 NP	7.30	28.37	18.03	0.99	29.61	101.14	5.88	0.99
CTA-CeO_2(0.64)	13.47	32.02	3.88	0.99	39.35	17.95	5.39	0.99

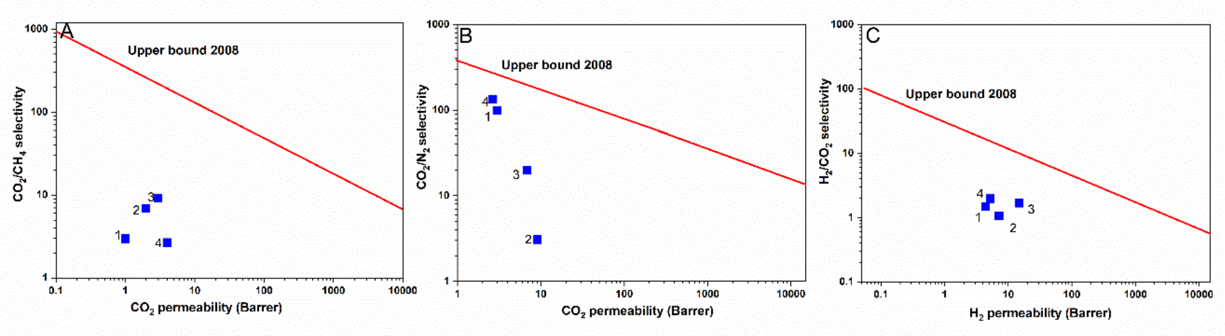

Figure S9. Robeson upper bound comparison for the different gas pairs in this study. The number in each symbol indicate the MMM samples: 1) CTA, 2) CTA-CeO_2 (0.32), 3) CTA-CeO_2 (0.64), and 4) CTA-CeO_2 (0.9).

Publisher's Note: MDPI stays neutral with regard to jurisdictional claims in published maps and institutional affiliations.

Copyright: © 2021 by the authors. Submitted for possible open access publication under the terms and conditions of the Creative Commons Attribution (CC BY) license (https://creativecommons.org/licenses/by/4.0/).
